# The role of fluconazole in the regulation of fatty acid and unsaponifiable matter biosynthesis in *Schizochytrium* sp. MYA 1381

**DOI:** 10.1186/s12866-019-1622-4

**Published:** 2019-11-15

**Authors:** Jun Li, Hao Zhou, Xueshan Pan, Zhipeng Li, Yinghua Lu, Ning He, Tong Meng, Chuanyi Yao, Cuixue Chen, Xueping Ling

**Affiliations:** 1 Department of Chemical and Biochemical Engineering, College of Chemistry and Chemical Engineering, Xiamen University, Xiamen, 361005 People’s Republic of China; 2 Fujian Collaborative Innovation Center for Exploitation and Utilization of Marine Biological Resources, Xiamen, Fujian People’s Republic of China; 3 The Key Lab for Synthetic Biotechnology of Xiamen City, Xiamen University, Xiamen, 361005 People’s Republic of China

**Keywords:** *Schizochytrium*, Fluconazole, Unsaponifiable matter, Metabolic analysis, Fatty acids.

## Abstract

**Background:**

*Schizochytrium* has been widely used in industry for synthesizing polyunsaturated fatty acids (PUFAs), especially docosahexaenoic acid (DHA). However, unclear biosynthesis pathway of PUFAs inhibits further production of the *Schizochytrium*. Unsaponifiable matter (UM) from mevalonate pathway is crucial to cell growth and intracellular metabolism in all higher eukaryotes and microalgae. Therefore, regulation of UM biosynthesis in *Schizochytrium* may have important effects on fatty acids synthesis. Moreover, it is well known that UMs, such as squalene and β-carotene, are of great commercial value. Thus, regulating UM biosynthesis may also allow for an increased valuation of *Schizochytrium*.

**Results:**

To investigate the correlation of UM biosynthesis with fatty acids accumulation in *Schizochytrium,* fluconazole was used to block the sterols pathway. The addition of 60 mg/L fluconazole at 48 h increased the total lipids (TLs) at 96 h by 16% without affecting cell growth, which was accompanied by remarkable changes in UMs and NADPH. Cholesterol content was reduced by 8%, and the squalene content improved by 45% at 72 h, which demonstrated fluconazole’s role in inhibiting squalene flow to cholesterol. As another typical UM with antioxidant capacity, the β-carotene production was increased by 53% at 96 h. The increase of squalene and β-carotene could boost intracellular oxidation resistance to protect fatty acids from oxidation. The NADPH was found to be 33% higher than that of the control at 96 h, which meant that the cells had more reducing power for fatty acid synthesis. Metabolic analysis further confirmed that regulation of sterols was closely related to glucose absorption, pigment biosynthesis and fatty acid production in *Schizochytrium*.

**Conclusion:**

This work first reported the effect of UM biosynthesis on fatty acid accumulation in *Schizochytrium.* The UM was found to affect fatty acid biosynthesis by changing cell membrane function, intracellular antioxidation and reducing power. We believe that this work provides valuable insights in improving PUFA and other valuable matters in microalgae.

## Background

*Schizochytrium* sp., a kind of marine microalga, has drawn increasing attention for synthesizing significant amounts of total lipids rich in PUFAs, especially docosahexaenoic acid (DHA,22:6) [1]. DHA is an ω-3 PUFA and plays an important role in promoting mental development in infants and preventing cardiovascular diseases [2]. Novel sources of ω-3 PUFA can be green manufactured from *Schizochytrium* sp., which could also eliminate many problems such as bad taste from the traditional source of fish oil [3]. *Schizochytrium* sp. was reported to synthesize PUFAs through polyketide synthase (PKS) and fatty acid synthase (FAS) pathways [4]. In recent years, most studies attempted to elucidate the PUFA synthesis pathway based on regulation of key genes related to biosynthesis in order to improve PUFA production [5–8]. However, in these studies, genetic engineering methods such as use of genetically engineered microorganisms are employed. It should be noted that, the genetically engineered microorganisms have been widely questioned in the food field [9]. More importantly, most studies were hindered due to the unknown mechanism of PUFA synthesis in *Schizochytrium* sp. In addition, PUFA synthesis is also affected by other related metabolic pathways because of the complexity of metabolism. Therefore, regulating other related metabolic pathways may be an effective method to increase PUFAs production [10].

**Fig. 1:**
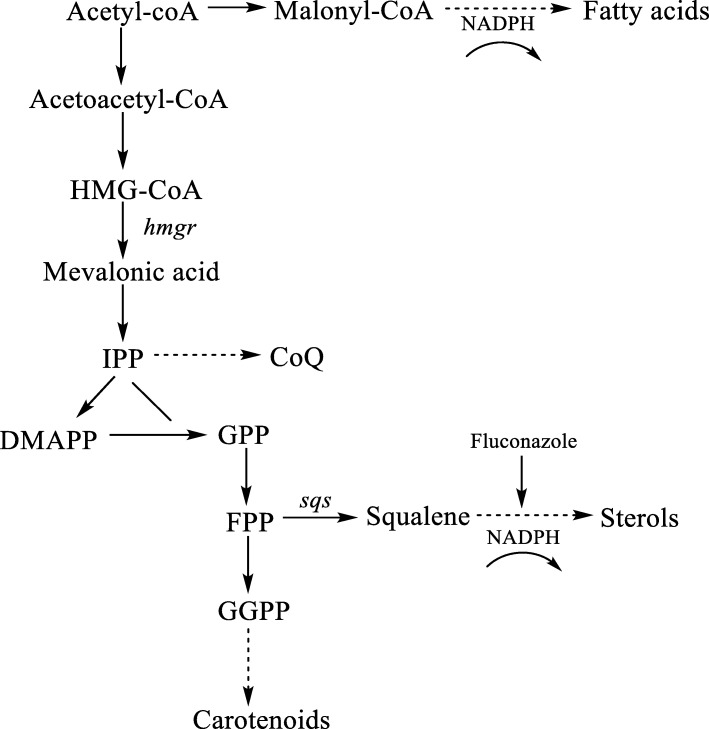
Summary of the carotenoid, fatty acid, and sterol biosynthetic pathways in *Schizochytrium* sp.

Ren et al. found that the content of PUFAs has a positive correlation with UM concentration [11]. Compared to fatty acids, UM refers to lipid-soluble matter that could not undergo saponification including pigments, squalene and sterols, which are isoprenoid compounds synthesized by the mevalonate acid (MVA) pathway (Fig. 1). As a pathway of UM synthesis, the MVA plays an important role in cell growth and intracellular metabolism and exists in all higher eukaryotes and many viruses [12]. In the initial step, two molecules of acetyl-CoA (which is also the initial substrate of fatty acid synthesis) undergo condensation to yield acetoacetyl-CoA, which is subsequently converted to 3-hydroxy-3-methylglutaryl-CoA (HMG-CoA) by the gene product of 3-Hydroxy-3-methylglutaryl-coenzyme A synthase (*hmgs*). HMG-CoA is further converted to MVA by the gene product of *hmgr*, which is the rate-limiting step of the MVA pathway [13]. Then, MVA is further converted to isopentenyl diphosphate (IPP), which is the precursor of coenzyme Q (CoQ) a hydrogen carrier in the electron transport chain and the necessary ingredients for mitochondrial ATP synthesis [14]. Farnesyl diphosphate (FPP) is synthesized from IPP in several steps [15]. FPP is a crucial molecule in the MVA pathway. Specifically, it is condensed directly by squalene synthase (*sqs*) to squalene, which is subsequently converted to sterols [16]. The other FPP is condensed to carotenoids through a series of condensation, dehydrogenation and cyclization reactions.

The UM from MVA pathway is important to intracellular metabolism. It is well known that both sterols and fatty acids are the main components of the cell membrane, which has a significant influence on fatty acid synthesis [17]. Bernsdorff et al. found out that different sterols have different effects on the regulation of the cell membrane due to their distinctive molecular structures [18]. Squalene is a natural antioxidant that is capable of removing excess free radicals in cells, thus protecting lipids from peroxidation [19, 20]. On the other hand, carotenes have a close relationship with intracellular oxidative stress, which has a significant relationship with cell growth and lipid accumulation [21, 22]. Rice et al. reported that the gene *mga*2, which encodes an important regulator of unsaturated fatty acid production in *Saccharomyces cerevisiae*, affected transcription and expression of the *erg*1 gene, which encodes squalene oxidase. The *mga*2 deletion strain had relatively elevated amounts of squalene compared to wild-type cells [23]. This UM is crucial to the cell growth and fatty acid biosynthesis. Therefore, regulation of UM biosynthesis in *Schizochytrium* sp. may have important effects on the synthesis of fatty acids. Moreover, it is well known that UM, such as squalene and β-carotene, may be of great commercial value. Thus, regulating UM biosynthesis may also allow for an increased valuation of *Schizochytrium.*

Fluconazole, a type of antifungal agent, blocks the sterol pathway by inhibiting lanosterol 14α-demethylase activity [24]. At present, fluconazole is mainly used as a drug in the treatment of fungal infections. Miao et al. found that blocking the sterol pathway with 60 mg/L fluconazole could increase astaxanthin content to 5-fold higher than that of the control in *P. rhodozyma* [25], which indicates that fluconazole could be used to regulate UM metabolism. Based on this result, fluconazole was added to the medium in this study to investigate its effects on UM and fatty acid biosynthesis in *Schizochytrium* sp. MYA 1381. Our work aims to illuminate the relationship between fatty acid and UM biosynthesis in providing a strategy for regulating the production of PUFAs in *Schizochytrium* and also explore additional value of the *Schizochytrium*.

## Materials and methods

### Medium, strains, and culture conditions

*Schizochytrium* sp. MYA1381 was obtained from the American Type Culture Collection (USA) and maintained on seed broth agar plates. The fermentation and seed broths were the same as those used in our previous study [26]. After three generations of cultivation, the seed culture (4% v/v) was then transferred to a 250 mL flask containing 100 mL fermentation broth and was incubated at 28 °C and 200 rpm for 120 h or more in the dark. Fluconazole (60 mg/L) was dissolved in methanol and added to the fermentation broth. Each treatment was repeated three times. Samples from the control and fluconazole groups were collected every 24 h until the total fermentation time reached 120 h. The biomass, lipid content, fatty acid and metabolic profile were analyzed as described below.

### Determination of dry cell weight (DCW) and glucose

One milliliter of broth was transferred to a pre-weighed centrifuge tube and then centrifuged at 8000×g for 2 min. The cell pellet was washed twice with distilled water and lyophilized to a constant weight at − 50 °C for approximately 24 h. DCW was then weighed. The supernatant from the centrifugation was collected to measure the residual glucose concentration. The glucose concentration was determined by the 3,5-dinitrosalicylic acid (DNS) method [27].

### Lipid extraction and fatty acid composition analysis

Three milliliters of fermentation broth was mixed with 4 mL HCl (12 N) and incubated in a water bath at 65 °C for 45 min. TLs from the mixture were extracted four times with 3 mL n-hexane, and then the lipid extract was purified and dried by evaporation. Total fatty acids (TFA) production was calculated by subtracting UM from TLs, where UM was isolated from lipids by saponification [28]. The fatty acid methyl esters (FAMEs) preparation and analysis were performed according to our previous study [26]_._

### Isolation and analysis of cholesterol, squalene and β-carotene

The UM isolated from lipids by saponification was prepared for measuring cholesterol and squalene. Cholesterol measurements were performed according to the GB/T 5009.128–2003. Squalene was analyzed by a GC system (Agilent GC 7890, USA) according to a previous study [29]. Because β-carotene is easily oxidized, it was extracted from cells to prevent saponification. Cells were harvested by centrifugation at 8000×g and washed with deionized water and then suspended with 5 mL petroleum ether/acetone mixture (7:3, vol/vol), followed by homogenate with a high- pressure homogenizer to release intracellular metabolites. After centrifugation, the supernatant was analyzed using high-performance liquid chromatography to measure the β-carotene content according to our previous study [30].

### Determination of G6PDH and NADPH

The glucose-6-phosphate dehydrogenase (G6PDH) activities were determined according to a previous study with minor modifications [31]. Cells were harvested by centrifugation at 4000×g for 2 min. The pellet was suspended in PBS buffer and then broken by a high-pressure homogenizer. The disrupted cell suspension was centrifuged at 12000×g for 10 min at 4 °C, and the supernatant was used for G6PDH activities. G6PDH was determined using continuous spectrophotometric assays following the increase of NADPH at 340 nm, and the enzyme activity was defined as the reducing amount of NADP^+^ (nmol) catalyzed by the enzyme solution with 1 mg of protein in 1 min (nmol min^− 1^ (mg protein)^− 1^). The NADPH content was measured by an NADP/NADPH quantification kit (Sigma). The protein concentrations of enzyme solutions were determined according to the method of Bradford [32].

### Preparation of metabolome samples

Cells from the control and fluconazole groups were collected every 24 h from 72 h to 120 h. The metabolome samples of *Schizochytrium* were prepared according to the procedures of Yu et al. with minor modifications [33]. Five milliliters of the samples from different time points were quickly harvested and immediately mixed with 5 mL of prechilled pure methanol (− 40 °C, v/v) to quench the culture. The quenched cells were washed twice with 5 mL cold physiological saline (0.9% of sodium chloride solution) and ground into a fine powder with liquid nitrogen. For extraction, cell powder (approximately 0.15 g) was transferred into a 1.5 mL Eppendorf tube and then extracted twice with 0.5 mL of prechilled methanol (− 40 °C). 1 mL of sample obtained above and 5 μL of internal standard (heptadecanoic acid in n-hexane, 16 mg/mL) were mixed and dried in a vacuum centrifuge dryer. Sample derivatization procedures were performed according to the method of Yu et al. [34].

### GC-MS analysis and data analysis of metabolomics

The sample was analyzed by GC-MS using an Agilent 7890-5975C GC-MS solution system (Agilent, Sacramento, USA) with an hp-5 capillary column (30 m × 0.25 mm, 0.25 μm film thickness; Agilent J.W. Scientific, Folsom, USA). One microliter of sample was injected into the DB-5MS capillary column coated with 5% phenyl and 95% methylpoly siloxane in split-less mode. The column temperature was held at 70 °C for 2 min and then increased to 290 °C at a rate of 8 °C/min and held for 3 min. Helium was used as a carrier gas, and the flow was constant at 1 mL/min. The transfer line and ion source temperatures were 280 °C and 250 °C, respectively. The mass scan range was 50–600 *m/z*. A supervised partial least-squares discriminant analysis (PLS-DA) was subsequently performed to identify the metabolites contributing to differences between the control group and the fluconazole group. A metabolite with a variable influence on the projection value (VIP) higher than 1 indicates a significant contribution to the separation of groups in PL-SDA models.

## Results and discussion

### Effects of fluconazole on cell growth, fatty acid and DHA production in *Schizochytrium*

**Fig. 2:**
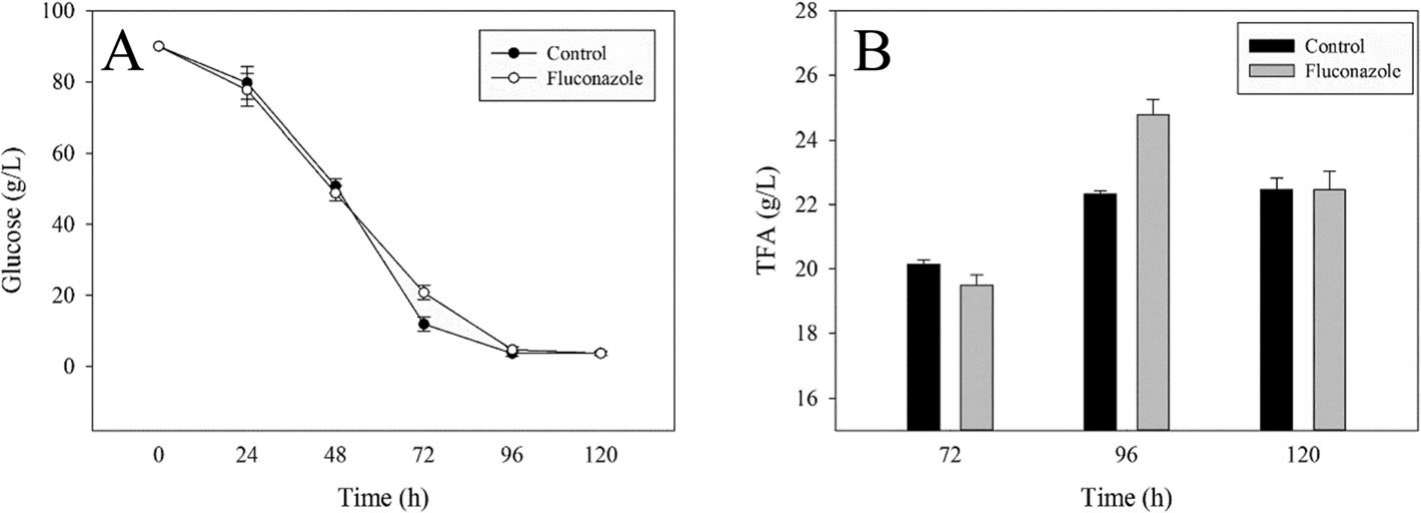
Residual glucose in the culture medium (**a**) and effect of adding 60 mg/L fluconazole at 48 h on TFA (**b**) in *Schizochytrium*. All data are the means of three replicates; vertical bars represent error bars with the value equal to the standard error of the mean

In this study, various fluconazole concentrations (0, 20, 40, 60, and 80 mg/L) were added at a culture time of 24 h to examine the effect of fluconazole on *Schizochytrium*. Compared with the control group (0 mg/L), the addition of fluconazole had little effect on cell growth. On the other hand, the fluconazole caused an increase in total lipids (TLs) and DHA yields at 96 h. Specifically, TLs and DHA contents (%DCW) increased to 24.5 g/L and 5.6 g/L, respectively, when 60 mg/L fluconazole was added (Table 1). To explore the influence of fluconazole on *Schizochytrium*, 60 mg/L fluconazole was added to the medium at three cultivation stages (0, 24 and 48 h). As shown in Table 2, the DCW had no obvious difference in all groups, meanwhile the TLs had a specific increase in three fluconazole groups. After the addition of 60 mg/L fluconazole at 48 h, the TLs increased to a maximum value of 25.3 g/L, which is 16% higher than that of control at 96 h, and were consequently chosen for further experiments. To investigate the effects of fluconazole on cell growth and fatty acid biosynthesis during the whole fermentation process, the TFA, DCW and the residual glucose in the culture medium were monitored from 72 to 120 h after addition of fluconazole at 48 h. From the results obtained, there were no obvious difference in biomass and TFA composition (data not shown). On the other hand, more residual glucose was observed in the fluconazole group than in the control group at 72 h (Fig. 2a). The observation is attributed to attenuation of glucose absorption from 48 h to 72 h due to the addition. Interestingly, TFA production in the fluconazole group began to increase after 72 h to a maximum of 25 g/L at 96 h, which is higher than that of the control group at 96 h. However, the increase maintained only for a short period of time. After the 96 h, the TFA yield started to decrease and reached the same value as the control at 120 h. Combined with the change in the residual glucose, the fluconazole added at the 48 h influenced the properties of the cell membrane by regulating sterol biosynthesis and the MVA pathway, thus attenuating the absorption of glucose and promoting fatty acid accumulation. To figure out the mechanism, some metabolites of the MVA pathway were observed in the following experiments.

**Table 1:** Effects of different concentrations of fluconazole on dry cell weight (DCW), total lipids (TLs), DHA yield and DHA/TFA at 96 h in *Schizochytrium*. Values are presented as the mean ± standard deviation (*n* = 3)

	Fluconazole concentration (mg/L)				
	0	20	40	60	80
Dry Cell Weight (DCW) (g/L)	43.6 ± 0.1	42.5 ± 0.5	41.8 ± 0.3	41.7 ± 0.1	43.3 ± 0.6
Total Lipids (TLs) (g/L)	21.1 ± 0.6	24.3 ± 0.2	23.4 ± 1.2	24.5 ± 0.1	22.4 ± 1.1
DHA (g/L)	5.0 ± 0.3	5.5 ± 0.1	5.4 ± 0.4	5.6 ± 0.1	5.2 ± 0.4
DHA/TFA	24 ± 0.2	23 ± 0.2	23 ± 0.1	23 ± 0.1	23 ± 0.7

**Table 2:** Effects of 60 mg/L fluconazole added at different cultivation time on dry cell weight (DCW), total lipids (TLs), DHA and DHA/TFA in *Schizochytrium*. Values are presented as the mean ± standard deviation (n = 3)

	Control	Addition time of fluconazole		
		0 h	24 h	48 h
Dry Cell Weight (DCW) (g/L)	43.8 ± 1.1	42.9 ± 1.3	42.0 ± 0.8	43.2 ± 1.0
Total Lipids (TLs) (g/L)	21.8 ± 0.5	22.2 ± 1.0	24.0 ± 0.9	25.3 ± 0.6
DHA (g/L)	5.2 ± 0.3	3.6 ± 0.1	5.5 ± 0.4	5.7 ± 0.1
DHA/TFA	24 ± 0.1	16 ± 1.1	23 ± 0.1	22 ± 0.0

### Influences of fluconazole on UM

Cholesterol, squalene and β-carotene are the three main kinds of UM in *Schizochytrium* sp*.* [11]. Based on the results shown in Fig. 2, the contents of cholesterol, squalene and β-carotene at 72, 96 and 120 h were measured to investigate the effect of fluconazole addition at 48 h on UM biosynthesis with an aim of determining UM and fatty acid biosynthesis relationship.

**Fig. 3:**
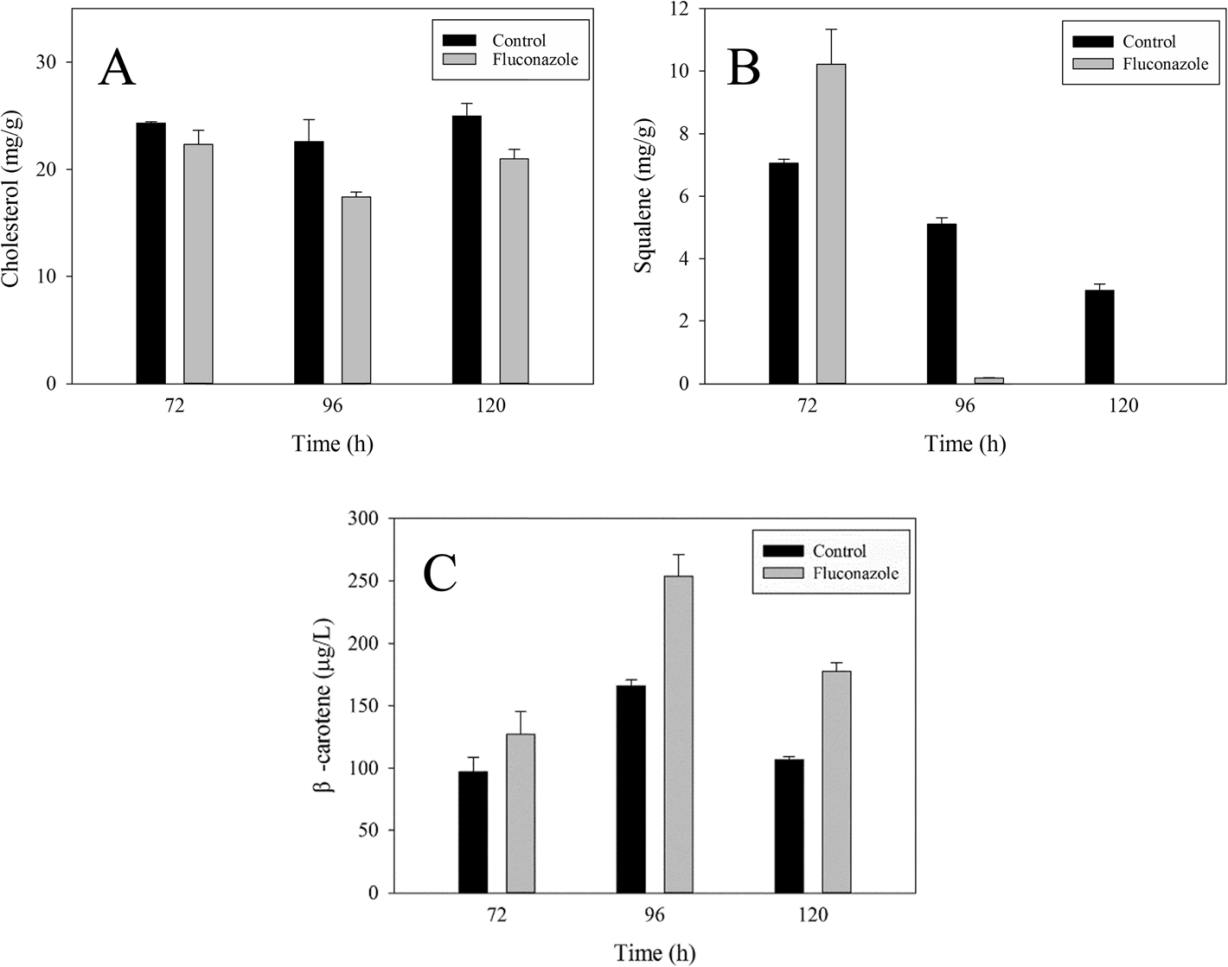
Effect of fluconazole on cholesterol (**a**), squalene (**b**) and β-carotene (**c**). All data are the means of three replicates; vertical bars represent error bars with the value equal to the standard error of the mean

As shown in Fig. 3a, the cholesterol content (mg/g lipid) in the fluconazole group was lower than that in the control group at three cultivation times, a demonstration of fluconazole role in inhibiting sterol synthesis. Cholesterol is known to be the main component in the cell membrane and is crucial for the absorption of nutrients. Therefore, a decrease in cholesterol content could account for more residual glucose in the fluconazole group (Fig. 2a). Furthermore, blocking sterol biosynthesis could save more NADPH for fatty acid biosynthesis (Fig. 1), which may cause higher TFA production in the fluconazole group.

The squalene content (mg/g lipid) in the fluconazole group was 45% higher than that of the control group at 72 h, after which it dropped quickly to near zero at 96 h and to zero at 120 h (Fig. 3b). Note that, the point of fluconazole inhibition was found to be between squalene and cholesterol. As shown in Fig. 3a, the inhibition of the sterol pathway by fluconazole resulted in over-accumulation of squalene for a short period of time, a finding that is consistent with Cai-Jun Yue [35]. Note also that, the change in squalene content showed a positive relationship with TFA production. It is known that squalene is an essential natural antioxidant that protects cells from free radicals and reactive oxygen species (ROS), it also plays a major role in preventing oxidative stresses [36]. Therefore, when more squalene accumulates at 72 h in the fluconazole group, it may protect fatty acids from being preferentially oxidized from 72 h to 96 h, leading to consumption of squalene in large quantities and accumulation of fatty acids at 96 h. With the utilization of squalene, TFA production decreased from 96 h to 120 h (Fig. 2b).

After the addition of fluconazole in the medium, the β-carotene production was significantly enhanced than that in the control group (Fig. 3c). In particular, the β-carotene content in the fluconazole group was 253.5 μg/L at 96 h, which was 53% higher than that of the control. As shown in Fig. 1, shows β-carotene biosynthesis shares FPP with the squalene pathway. Therefore, by blocking the sterol pathway by fluconazole, squalene over-accumulates, resulting in more FPPs that are channeled to carotenoid biosynthesis. As a kind of lipid-soluble orange pigment, β-carotene is another natural antioxidant that could protect intracellular protein, DNA and lipids from oxidation by eliminating ROS [21]. Therefore, more β-carotene accumulation is favorable for fatty acid accumulation. These results imply that UM affects fatty acid biosynthesis by changing cell membrane function, ROS level and antioxidation. It is also deduced that the metabolic flux of NADPH is regulated by the inhibition of the sterol pathway with fluconazole, which influences fatty acid biosynthesis. To confirm this inference, the intracellular NADPH content was measured in the following experiments.

### Influence of fluconazole on G6PDH activity and NADPH content

**Fig. 4:**
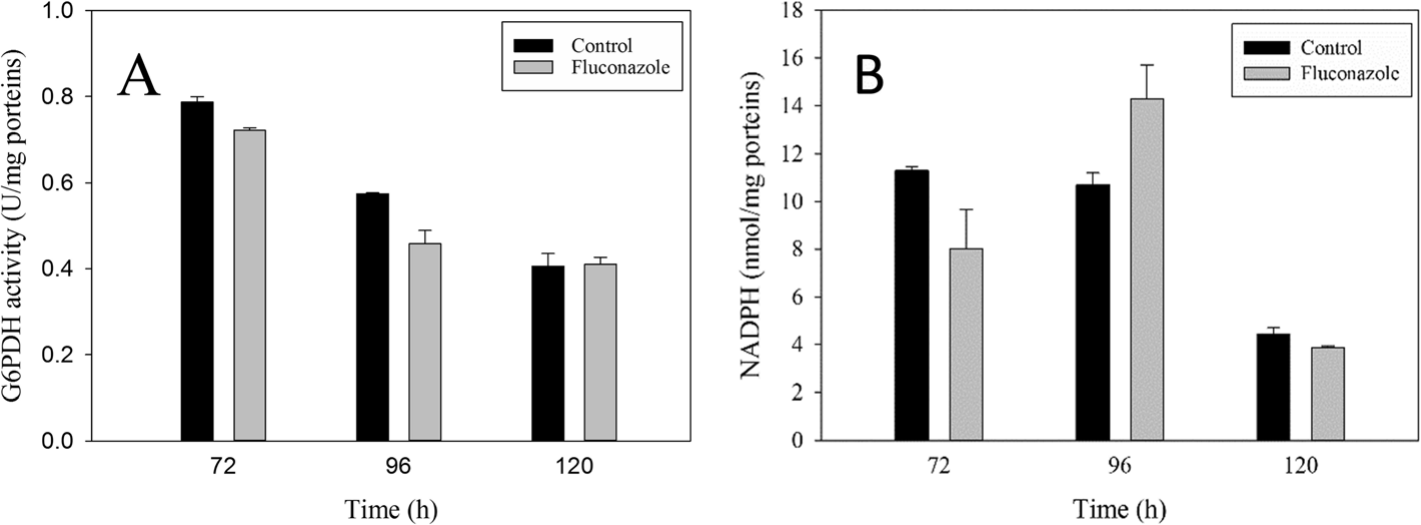
Effect of fluconazole on G6PDH activity (**a**) and NADPH content (**b**). All data are the means of three replicates; vertical bars represent error bars with the value equal to the standard error of the mean

NADPH is an essential reducing agent for anabolism and one of the most important cofactors for the synthesis of fatty acid and UM. Moreover, it is also necessary forantioxidant system [37]. NADPH is mainly synthesized through the pentose phosphate pathway (PPP) and the TCA pathway [6]. On the other hand, glucose-6-phosphate dehydrogenase (G6PDH) and malic enzyme (ME) are key enzymes of the two pathways producing NADPH, respectively. Therefore, the addition of 4 g/L malic acid at the rapid lipid accumulation stage increased total lipid by 15% by increasing NADPH supply [38]. In particular, G6PDH plays a role in controlling carbon flux and plays a critical role in providing reducing power in microalgae [6, 39]. Thus, G6PDH activity and intracellular NADPH content were checked. As shown in Fig. 4a, the G6PDH activities were similar in both groups, which meant that the biosynthesis of NADPH was not influenced by fluconazole. On the other hand, the intracellular NADPH content showed an interesting change. In the fluconazole group, the NADPH content was 41% lower than that of the control at 72 h, while it increased to 13.7 nmol/mg protein at 96 h, which was 33% higher than that of the control (Fig. 4b). In addition, at 120 h, the NADPH content was similar to that of the control group, which meant that the metabolic flux of NADPH was regulated. It is well known that NADPH is consumed in the cholesterol biosynthesis pathway. Therefore, inhibition of cholesterol production by fluconazole inhibits, some NADPH for sterol synthesis is saved and can be shifted to fatty acid biosynthesis. Meanwhile, more squalene was observed at 72 h in the fluconazole group (Fig. 3b). As well know, squalene can decrease ROS levels by eliminating oxygen free radicals. Sun et al. pointed out that the squalene content of endpoint strains was reduced by 63% in *Schizochytrium* sp. under high oxygen supply conditions [40]. Therefore, a higher squalene content may imply lower intracellular ROS levels in the fluconazole group at 72 h, where more NADPH was released from the antioxidant system from 72 h to 96 h, which could also explain why more NADPH was observed at 96 h in the fluconazole group [22].

### Metabolomics analysis

#### Comparative metabolite profile of with and without fluconazole treatment

**Fig. 5:**
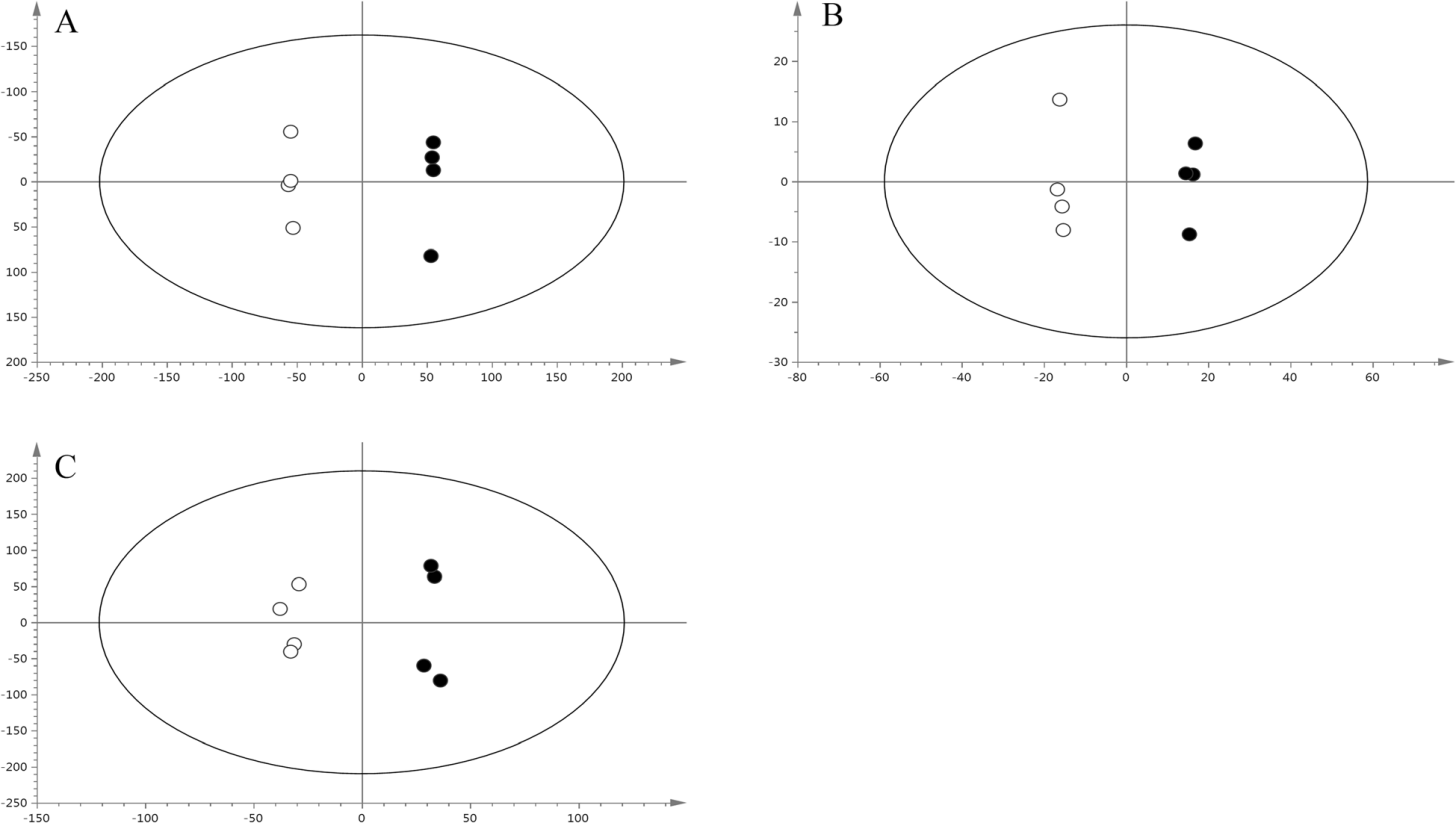
PLS-DA derived plots for the control group (open symbols) and the fluconazole-treated group (full symbols). (**a**), (**b**), and (**c**) present PLS-DA derived plots for the control group and the fluconazole group at 72 h, 96 h, and 120 h, respectively

**Fig. 6:**
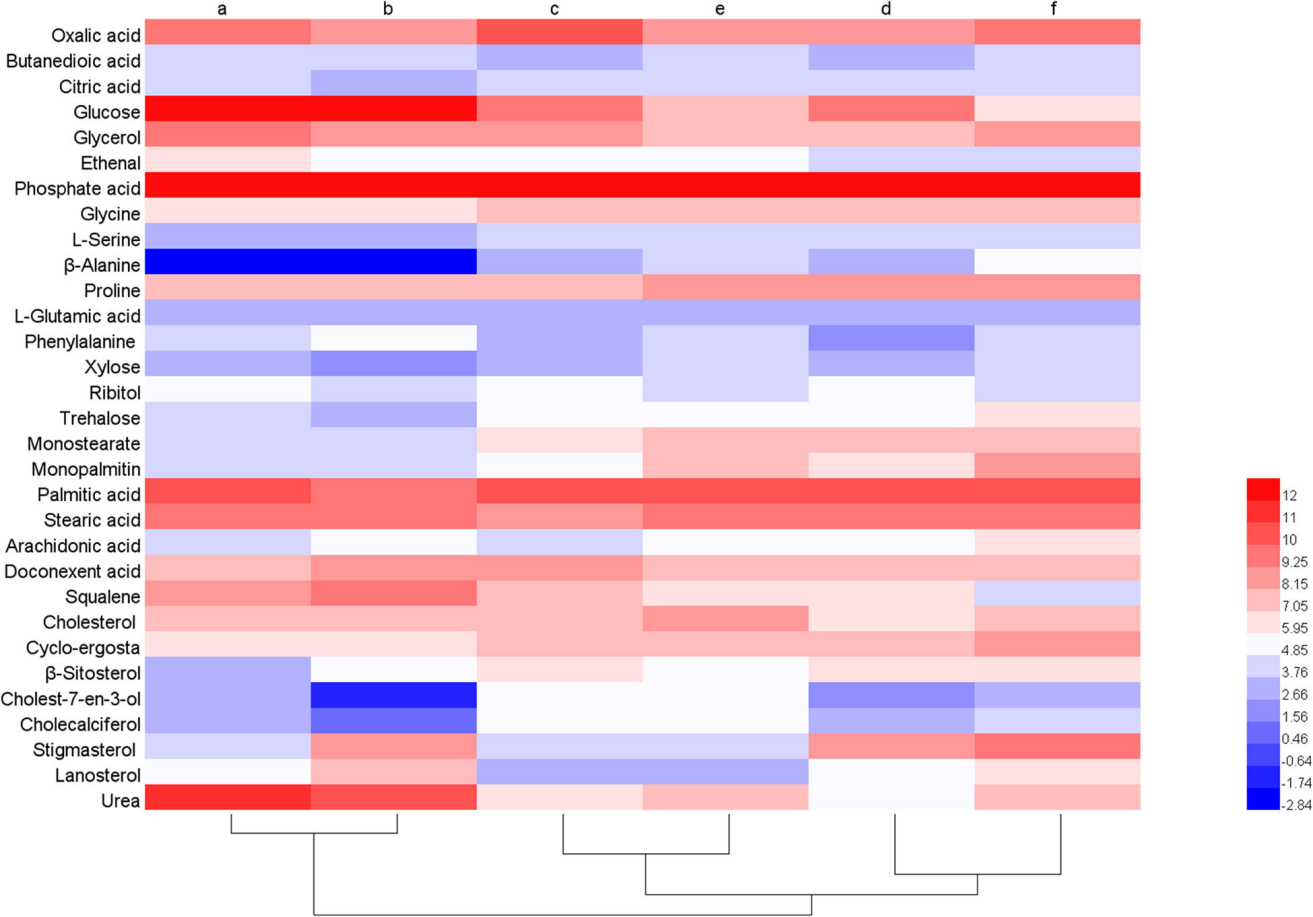
Clustering of the intracellular metabolites for the control group and the fluconazole group. (**a-f**) represent the control group at 72 h, the fluconazole group at 72 h, the control group at 96 h, the fluconazole group at 96 h, the control group at 120 h, and the fluconazole group at 120 h, respectively. The metabolite levels correspond to the rectangle colors. Red and blue represent higher and lower levels of the metabolites, respectively

Metabonomics was performed to analyze the intracellular metabolites in the presence of 60 mg/L fluconazole at 72, 96, and 120 h. As shown in Fig. 5, the PLS-DA pairwise comparisons showed a significant separation of metabolic profiles between the control group and the fluconazole group at 72 (A), 96 (B), and 120 h (C). A total of 31 kinds of intracellular metabolites were selected and identified with the aid of the NIST database (Table. 3). Furthermore, as shown in Fig. 6, a schematic diagram of the proposed metabolic pathways was mapped out to provide deep insight into the intracellular metabolism of *Schizochytrium* according to a previous study [33] and the Kyoto Encyclopedia of Genes and Genomes (KEGG).

**Table 3:** The metabolites content (μg/g DCW) responsible for responding to fluconazole in *Schizochytrium*

Metabolites	72 h		96 h		120 h	
	control	fluconazole	control	fluconazole	control	fluconazole
TCA cycle						
Oxalic acid	924.4 ± 22.7	557.3 ± 18.6*	1317.5 ± 114.3	597.8 ± 122.1*	575.9 ± 48.1	737.7 ± 33.3*
Butanedioic acid	21.4 ± 7.1	24.1 ± 11.1	11.3 ± 1.6	10.9 ± 2.4	21.1 ± 6.6	18.1 ± 5.3
Citric acid	22.6 ± 0.6	8.3 ± 1.1*	22.6 ± 9.9	13.6 ± 6.9*	26.9 ± 7.8	22.6 ± 8.3*
Glycolysis pathway						
Glucose	8503.2 ± 591.9	6838.7 ± 180.3*	654.1 ± 10.4	904.9 ± 18.4	154.2 ± 69.3	118.9 ± 48.3
Glycerol	633.5 ± 82.5	469.4 ± 54.5*	351.3 ± 14.9	226.7 ± 27.8*	227.5 ± 17.8	399.7 ± 1111.9*
Ethenal	75.7 ± 4.9	51.7 ± 9.8*	33.0 ± 0.7	25.3 ± 10.3*	163.3 ± 29.2	154.7 ± 31.5
Phosphate acid	12,811.2 ± 324.3	8389.3 ± 701.5	4413.8 ± 511.0	4622.4 ± 119.0*	10,915.5 ± 2687.4	9960.5 ± 4480.4
Amino acids						
Glycine	74.9 ± 11.6	74.1 ± 11.1*	234.5 ± 23.8	148.5 ± 9.8*	147.4 ± 70.0	163.9 ± 63.2
L-Serine	12.2 ± 3.2	10.1 ± 5.0	22.1 ± 1.1	18.6 ± 1.4*	4.4 ± 4.4	18.4 ± 8.2
β-Alanine	0.1 ± 0.0	0.3 ± 0.0*	7.4 ± 0.8	9.0 ± 1.4*	17.1 ± 4.4	29.8 ± 8.6*
Proline	225.0 ± 28.2	202.7 ± 22.8*	156.1 ± 55.1	322.9 ± 75.2*	427.1 ± 123.8	337.8 ± 40.7
L-Glutamic acid	7.1 ± 0.4	8.2 ± 1.9	10.2 ± 1.6	6.3 ± 5.0*	9.4 ± 1.6	8.9 ± 1.5
Phenylalanine	13.7 ± 0.7	29.0 ± 5.3*	10.8 ± 4.6	6.0 ± 4.5*	21.1 ± 2.6	14.6 ± 4.0
Pentose phosphate pathway						
Xylose	7.8 ± 1.0	5.6 ± 0.5*	10.6 ± 1.2	7.1 ± 11.4*	17.8 ± 3.5	22.5 ± 3.8
Ribitol	30.7 ± 1.2	20.8 ± 2.9*	54.1 ± 0.5	44.7 ± 23.4*	18.1 ± 5.3	20.9 ± 3.0
Trehalose	21.1 ± 7.7	11.9 ± 11.1*	42.6 ± 3.7	59.8 ± 6.1*	45.5 ± 5.0	69.2 ± 6.4
Fatty acids						
Monostearate	23.5 ± 16.1	21.5 ± 10.5*	127.2 ± 18.5	239.5 ± 48.2*	155.9 ± 29.0	242.6 ± 31.0*
Monopalmitin	17.7 ± 2.3*	17.6 ± 4.6	57.1 ± 9.0	83.2 ± 6.8*	268.0 ± 47.3	375.8 ± 60.3*
Palmitic acid	1166.0 ± 77.5	889.9 ± 119.5	1712.2 ± 113.8	1262.7 ± 86.2*	1188.5 ± 151.0	1224.8 ± 246.0
Stearic acid	1018.4 ± 107.2	648.4 ± 157.5*	593.5 ± 82.7	748.5 ± 72.6	703.9 ± 113.1	763.0 ± 109.9
Arachidonic acid	19.9 ± 4.1	33.8 ± 4.6*	28.5 ± 2.5	36.3 ± 2.4*	41.9 ± 4.1	61.5 ± 15.2*
Doconexent acid	237.5 ± 32.4	359.0 ± 26.9*	314.2 ± 33.6	267.0 ± 17.4	169.7 ± 31.5	271.5 ± 34.6*
Sterols						
Squalene	510.2 ± 33.8	610.6 ± 80.0*	161.4 ± 5.8	63.1 ± 0.3*	88.6 ± 10.7	16.8 ± 8.5*
Cholesterol	271.4 ± 40.9	202.9 ± 10.2*	246.8 ± 18.0	95.8 ± 2.9*	286.8 ± 28.3	254.2 ± 20.5
Cyclo-ergosta	120.8 ± 6.3	111.6 ± 7.9*	194.5 ± 18.8	204.4 ± 12.0	198.2 ± 28.9	287.5 ± 80.2*
β-Sitosterol	10.9 ± 2.5	47.8 ± 3.4*	86.3 ± 18.8	81.8 ± 4.7	51.1 ± 4.8	122.9 ± 32.4*
Cholest-7-en-3-ol	8.2 ± 0.1	0.6 ± 0.3*	51.0 ± 4.5	6.0 ± 0.4*	48.2 ± 7.0	8.4 ± 2.0*
Cholecalciferol	7.1 ± 0.4	2.2 ± 0.5*	30.5 ± 4.7	10.4 ± 0.3*	32.7 ± 9.7	16.1 ± 8.2*
Stigmasterol	15.0 ± 6.4	353.7 ± 29.9*	14.7 ± 3.7	483.9 ± 9.9*	19.9 ± 13.8	638.1 ± 108.3*
Lanosterol	47.0 ± 4.8	152.9 ± 2.9*	9.6 ± 0.7	37.9 ± 1.0*	9.2 ± 1.6	72.2 ± 25.7*
Others						
Urea	2103.6 ± 183.8	1248.0 ± 130.2*	76.2 ± 4.2	32.9 ± 18.6*	71.4 ± 13.2	59.5 ± 19.9

* *p* < 0.05

The data represents the contents of metabolites and are showed as the mean ± SD. All data are four of replicates

#### Fatty acid synthesis variation induced by fluconazole

Fatty acids mainly exist in the forms of triacylglycerol, monoacylglycerol, diacylglycerol, free fatty acids, and phospholipids in *Schizochytrium* sp. [41]. From the GC-MS results (Table 3), only the free fatty acids monostearate and monopalmitin were detected. Other existing forms of fatty acids, such as diacylglycerol and triglyceride, could not be detected in GC-MS given their large size. As shown in Table 3, the concentration of the free fatty acids, including arachidonic acid (ARA) and DHA, was elevated in the fluconazole group. Monostearate and monopalmitin were also elevated at 96 and 120 h. The concentration of free saturated fatty acids, such as palmitic acid and stearic acid, had hardly changed. Therefore, increase in free PUFAs and acylglycerol of saturated fatty acids mainly contributed to the increase in TFA (Fig. 2b). To our knowledge, PUFAs and sterols are important components of the cell membrane. Thus, when sterol biosynthesis is regulated, the biosynthesis and existential forms of PUFA are affected to balance the structural and dynamical properties of the cell membrane.

#### Metabolic pathways that are related to fatty acid variation induced by fluconazole

**Fig. 7:**
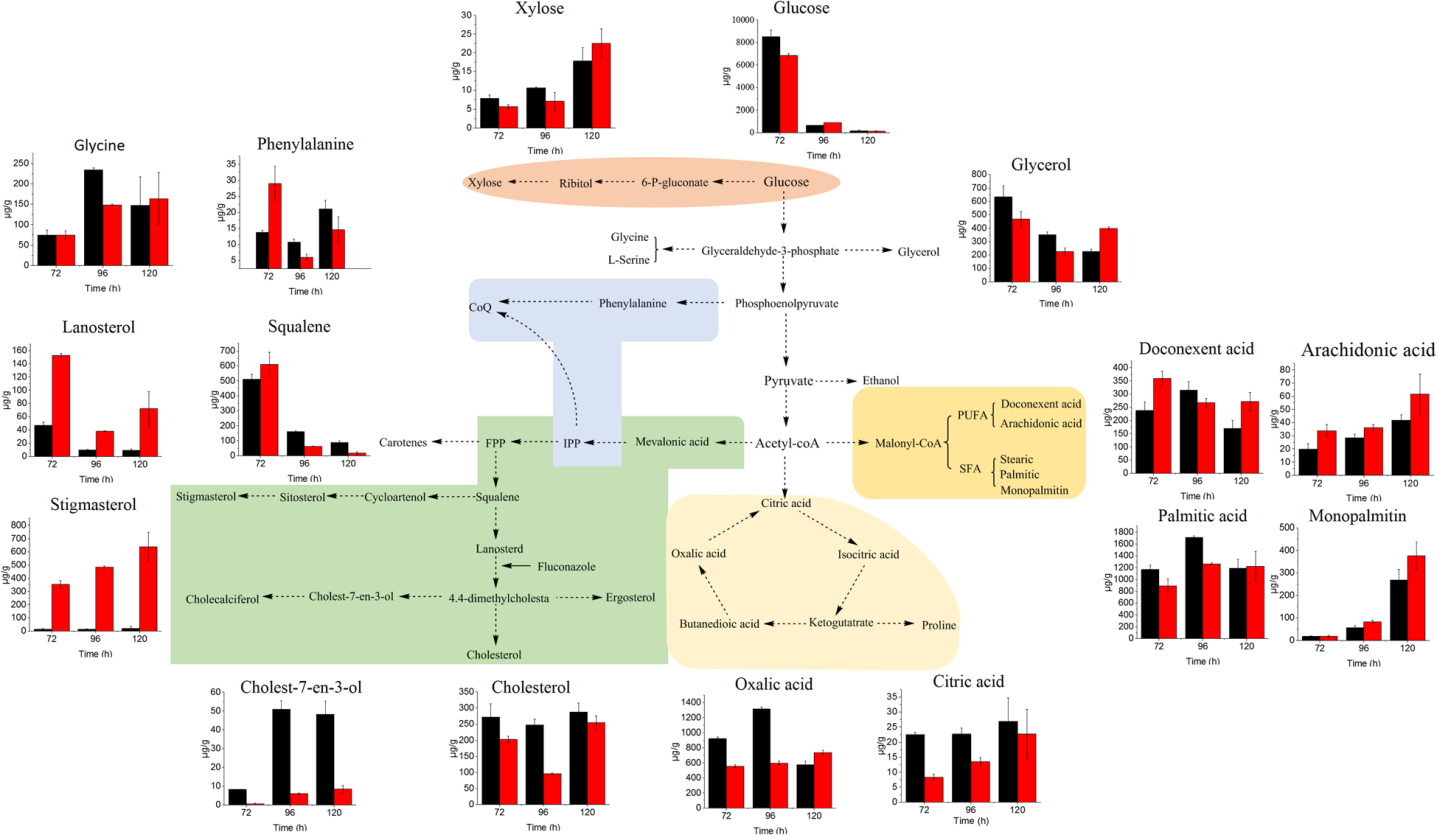
A schematic diagram of the proposed metabolic pathways in *Schizochytrium.* The black and red rectangles represent the metabolites in the control group and the fluconazole group, respectively

As shown in Table 3 and Fig. 6, the changes in cholesterol and squalene contents are in line with the results in Fig. 3a and Fig. 3b. Moreover, six other kinds of sterols, namely, lanosterol, ergosterol, cholest-7-en-3-ol, cholecalciferol, β-sitosterol and stigmasterol, were also observed by GC-MS. Due to inhibition of the sterol pathway, cholest-7-en-3-ol, cholesterol and cholecalciferol contents in the fluconazole group were much lower than those in the control group. As the substrate of 14α-demethylase, which was inhibited by fluconazole, the lanosterol content was significantly increased in the fluconazole group, which further resulted in the over-accumulation of squalene. The increased squalene as an antioxidant might scavenge intracellular ROS in *Schizochytrium* for lipid accumulation. It is reported that other exogenous additives such as ascorbic acid and sesamol increase the antioxidant capacity of cells for lipid accumulation [42]. On the other hand, it was observed that the content of ergosterol was not significantly affected by fluconazole. Ergosterol is a fungal sterol and is the most important sterol in cell membranes, where it is crucial for cell growth and maintaining morphology. Therefore, the ergosterol may preferentially be synthesized to maintain its activity when the sterol pathway is blocked in marine microalgae. Interestingly, stigmasterol and β-sitosterol were greatly increased. The observation is attributed to the prompt accumulation of squalene and channeling of more squalene into stigmasterol and β-sitosterol biosynthesis due to inhibition of lanosterol 14α-demethylase activity by fluconazole (Fig. 7) [43]. Stigmasterol and β-sitosterol are phytosterols, which are the most important components of cell membranes in plants. Compared with cholesterol, stigmasterol has one more double bond, which decreases the fluidity of the cell membrane [18]. This structural aspect could also account for the slow absorption of glucose in fluconazole group. Moreover, stigmasterol has the potential to treat ovarian, prostate, breast and colon cancers [44]; however, its biosynthesis and role have never been reported in marine microalgae. To the best of our knowledge, this is the first report on stigmasterol biosynthesis in marine microalgae. These findings may provide novel insight for future research on *Schizochytrium*.

The intracellular glucose content decreased in the fluconazole group due to reduction of cell membrane’s fluidity as a result of profile change of sterols in the cell membrane, thus retarding the absorption of glucose, as evident by more residual glucose in the culture medium of fluconazole group. An et al. and Saniewski et al. pointed out that sterol and PUFAs play a role in membrane fluidity and permeability, further improving the absorption function of the membrane [45, 46]. Li et al. also pointed out that MAT overexpression could accelerate glucose absorption by improving membrane fluidity and permeability, and finally enhance carbon flux to the PUFA synthesis [47]. It was also found that the trehalose content of the fluconazole group was less than that of the control group at 72 h (Table 3 and Fig. 6). The accumulation of trehalose has been associated with the oxidative stress response [48]. Therefore, this result confirmed our previous assumption that lower ROS level was caused by squalene at 72 h.

Acetyl-CoA is a key intermediate metabolite, from which the metabolic flux can enter the MVA pathway, fatty acid biosynthesis and TCA cycle (Fig. 7). Citric acid, oxalic acid, and L-glutamic acid contents were reduced in the fluconazole group at 96 h (Table 3 and Fig. 6), suggesting that more acetyl-CoA was channeled into fatty acid biosynthesis from 72 to 96 h, which was consistent with an increase in fatty acid observed at 96 h in the fluconazole group. Ruch et al. and Cheng et al. showed that acetyl-CoA was used to synthesize malonyl-ACP for fatty acid production [49, 50]. Li et al. pointed out that low citric acid, oxalic acid, and L-glutamic acid content favors fatty acid synthesis by weakening TCA cycle [47]. The decrease in glycine, L-serine and glycerol in the fluconazole group at 72 h and 96 h was due to the inhibited utilization of glucose, which reduced the synthesis of glyceraldehyde 3-phosphate. Geng et al. pointed out that some intermediate metabolites including glyceraldehyde-3-phosphate and phosphoenolpyruvic acid in glycolysis pathway reduced, which promoted the glycolytic pathway to synthesize pyruvate [51]. However, the content of phenylalanine in the fluconazole group was much higher than that in the control group at 72 h, but far less at 96 h and 120 h. This trend was the same as that of squalene and may be because more squalene and IPP accumulated when the sterol pathway was blocked. Both IPP and phenylalanine are substrates of coenzyme Q (CoQ) biosynthesis [14]. It is known that CoQ is combined with the mitochondrial membrane and is responsible for electron transport by transmitting hydrogen, which is necessary for mitochondrial ATP synthesis. Moreover, CoQ is capable of oxidation resistance by scavenging free radicals. Therefore, the accumulation of IPP induced an increase in phenylalanine production at 72 h to synthesize more CoQ to improve intracellular oxidation resistance, which might be another reason why more fatty acids accumulated from 72 h to 96 h.

## Conclusion

The present study investigated the effect of the inhibition of sterol biosynthesis by fluconazole on the UM and fatty acid biosynthesis in *Schizochytrium*. Blocking the sterol pathway could change the mobility of the cell membrane to postpone the absorption of glucose and shift more NADPH from the MVA pathway to fatty acid biosynthesis. In addition, when the sterol pathway was hindered, squalene over-accumulated and β-carotene biosynthesis was enhanced, which thus promoted fatty acid accumulation by improving oxidation resistance to protect fatty acids from oxidation. In summary, our work is the first to report the effect of the biosynthesis of UM on fatty acid accumulation in *Schizochytrium*, which provides valuable insights to guide future research in improving the production of PUFA and other valuable molecules, such as β-carotene, squalene and stigmasterol, in microalgae.

## Data Availability

All data generated or analyzed during this study are included in this manuscript.

## Abbreviations

DHA: Docosahexaenoic acid
MVA: Mevalonate acid
PUFA: Polyunsaturated fatty acid
TFA: Total fatty acid
TLs: Total lipids
UM: Unsaponifiable matter
